# The Associations between Maternal Serum Aspartame and Sucralose and Metabolic Health during Pregnancy

**DOI:** 10.3390/nu14235001

**Published:** 2022-11-24

**Authors:** Yu Liu, Xiaoyong Li, Yiming Wu, Qing Su, Li Qin, Jing Ma

**Affiliations:** 1 Department of Endocrinology and Metabolism, Renji Hospital, School of Medicine, Shanghai Jiao Tong University, 160 Pujian Road, Shanghai 200127, China; 2 Department of Endocrinology, Chongming Hospital Affiliated to Shanghai University of Health & Medicine Sciences (Chongming Branch of Xinhua Hospital), 25 Nanmen Road, Shanghai 202150, China; 3 Department of Endocrinology and Metabolism, Xinhua Hospital, Shanghai Jiao Tong University School of Medicine, 1665 Kongjiang Road, Shanghai 200092, China

**Keywords:** pregnancy, sweetener, metabolic health, gestational diabetes mellitus

## Abstract

Objective: We aimed to investigate the associations between maternal serum aspartame/sucralose levels and metabolic health during pregnancy. Methods: A nested population-based case-control study was conducted in 109 women with and without gestational diabetes mellitus (GDM). Serum aspartame and sucralose levels were assessed using an ultraperformance liquid chromatography coupled to a tandem mass spectrometry system. Results: We detected the presence of circulating aspartame and sucralose in all participants at fasting. No differences in serum aspartame or sucralose levels were observed between GDM and non-GDM groups. In the fully-adjusted linear regression models, serum aspartame levels were positively associated with insulin resistance index, total cholesterol, and LDL cholesterol. In the fully-adjusted logistic regression models, higher serum aspartame levels were positively associated with elevated HbA1c, insulin resistance, hypercholesterolemia, and hyper-LDL cholesterolemia. In the GDM group, the significant associations between higher serum aspartame levels and elevated HbA1c, insulin resistance, and hypo-HDL cholesterolemia persisted, while positive associations were found between higher serum aspartame levels and insulin resistance and hyper-LDL cholesterolemia in the non-GDM group. Serum sucralose levels were negatively associated with HbA1c. Conclusions: The study found that maternal serum aspartame levels were positively associated with insulin resistance index, total cholesterol, and LDL cholesterol during pregnancy. This finding provides the different effects of specific NNS on metabolic health during pregnancy.

## 1. Introduction

As “healthy” sugar substitutes with low-calorie content, non-nutritive sweeteners (NNSs) are used among all populations worldwide, including pregnant women. Cohort studies in Europe found that over 30% of pregnant women consumed artificially sweetened beverages during gestation [1]. Generally, NNS effects on human metabolism are thought to be non-harmful or even beneficial. More safety concerns arise from recent evidence showing the relationship between NNS exposure and dysmetabolism, particularly in pregnant women [2].

Current animal and human studies draw controversial conclusions about the effects of NNS exposure on metabolic disorders. In several animal studies, aspartame exposure resulted in body weight gain, obesity, glucose tolerance, and impaired insulin sensitivity [3,4,5]. However, a single dose of sucralose in the presence of carbohydrates could lower blood glucose in healthy subjects [6]. One of the proposed explanations for these conflicting results is the different sites of action on the sweet taste receptors (STRs) of each sweetener [7]. Therefore, to more precisely understand the effects of each NNS on metabolism, specific research on each sweetener is needed.

Previous human studies have commonly used questionnaires with NNS-containing foods to assess the amount of NNSs intake. Indeed, industrial food is not the only source of NNS exposure. A widespread and stable presence of NNS has been found in multiple personal care products, and surprisingly, in drinking water systems [8]. Emerging research reports that NNS are available in the fluids and organs of animals and human bodies [9,10,11]. Hence, measuring circulating NNS could be an alternative way to evaluate the association between NNS and metabolism. To our knowledge, literature regarding the associations between circulating NNS and metabolism was scarce.

The effects of NNSs during pregnancy on maternal metabolism have not been extensively studied. It has been reported that pregnant women with daily NNS beverage consumption gained more weight [1]. An animal study demonstrated that rats receiving aspartame exhibited more food intake during pregnancy [12].

Sucralose and aspartame are more widely used sweeteners, not only in diet soft drinks but also in water bodies [13]. Hence, the present study aimed to detect circulating aspartame and sucralose in women during pregnancy and evaluate the associations between serum aspartame and sucralose levels with glucose and lipid metabolism.

## 2. Methods

### 2.1. Participants

We conducted a nested case-control study of pregnant women from Chongming Hospital, affiliated to Shanghai University of Health and Medicine Sciences, from September 2019 to May 2021. With a confidence interval (CI) of 95%, power of 80%, and a prevalence of gestational diabetes (GDM) of 20% [14,15], we calculated a sample size of 385 participants using PASS 14 Power Analysis and Sample Size Software (NCSS, LLC. Kaysville, Utah). A completely random sampling with an effect size of 2 required 670 participants. Finally, 632 pregnant women were enrolled in the study at their first routine ultrasound examination (at 9–14 weeks of gestation). Specific exclusion criteria were presented as follows: (1) uncertain date of last menstrual period; (2) multiple pregnancies; (3) previous diagnosis of diabetes requiring treatment with medication before pregnancy; (4) secondary diabetes; (5) induction of pregnancy with the assistance of advanced reproductive technology.

In total, 113 of 632 participants were diagnosed with GDM. To maximize the statistical power, all women with GDM were selected as cases, except for 4 women missing blood samples. The final analysis included 109 cases and 109 controls matched for age and self-reported pre-pregnancy BMI. Group sample sizes of 109 cases and 109 controls achieved 100% power to show no difference in serum aspartame levels between the GDM and non-GDM groups using PASS 14 (Supplementary Figure S1). This study was approved by the institutional review board of Chongming Hospital Affiliated to Shanghai University of Health and Medicine Sciences (project number CMEC-2021-KT-30). Each participant signed a consent form.

### 2.2. Study Visit

Enrolled women underwent four visits during the perinatal period. Three face-to-face interviews were undertaken at 9–14, 24–32, and 34–36 weeks. The fourth visit was a phone interview completed one month before the due date.

Information on name, age, pre-pregnancy weight, gravidity and parity times, family history of diabetes, and history of chronic diseases was obtained during the first visit at 9–14 weeks gestation. Then, a comprehensive examination including a detailed questionnaire, anthropometric measurements, and biochemical evaluation was administered at 24–32 weeks gestation but as close to 28 weeks as possible. A 75-g oral glucose tolerance test (OGTT) was performed on the morning after at least 3 days of usual lifestyle and fasting for 8–10 hrs. Anthropometric measurements, including height, body weight, and blood pressure, were measured in the second trimester before OGTT. We calculated BMI as the weight divided by the squared height (kg/m^2^). We calculated maternal mean arterial pressure (MAP) as 1/3(SBP) + 2/3(DBP) [16]. Simple anthropometric measurements were administered between 34 and 36 weeks. Multiple attempts were made to contact eligible participants one month before the due date. Maternal and child outcomes were collected through a phone view.

### 2.3. Laboratory Measurements

Fasting, 1-h, and 2-h plasma glucose levels after glucose load were measured using the glucose oxidase method (ADVIA-1650 Chemistry System, Bayer, Leverkusen, Germany). HbA1c was measured via high-performance liquid chromatography (BIO-RAD, Laboratories, Hercules, CA, USA). Serum 1-h and 2-h insulin levels were measured using a chemiluminescent immunoassay (ARCHITECT ci16200 analyzer, Abbott Laboratories, Chicago, IL, USA). Homeostasis model assessment for insulin resistance (HOMA-IR) was calculated as follows: FPG (mmol/l) × FINS (mU/L)/22.5 [17,18]. Matsuda insulin sensitivity index (ISI) derived from OGTT was calculated as [10,000/sqrt (FPG × FINS × mean glucose × mean insulin) [19]. HOMA-β, reflecting the function of pancreatic β-cells, was calculated as [FINS × 20/(FPG-3.5)] [17]. Fasting triglycerides, total cholesterol, LDL cholesterol, and HDL cholesterol concentrations were measured using an automatic biochemical analyzer (AU5800; Beckman Coulter, Brea, CA, USA). Maternal serum samples during OGTT were immediately placed in an ice water slurry, processed within 60 min of collection, and stored at −80 °C until being used for circulating aspartame and sucralose tests.

### 2.4. Serum Aspartame and Sucralose Level Measurements

#### 2.4.1. Sample Preparation

Fasting serum specimens were collected during OGTT and stored at −80 °C until analyzed. Samples were thawed on ice at 4 °C before preparation. Pipettes of serum 80 (μL) were added into a 1.5 mL Eppendorf tube, with 400 μL pre-cooled methanol, and were then shaken at 1200 rpm for 20 min at 10 °C (MSC-100, Allsheng Instruments, Co., Ltd., Hangzhou, China). Then, the mixture was centrifuged at 18,000× *g* for 20 min at 4 °C (Microfuge 20R, Beckman Coulter, Inc., Indianapolis, IN, USA), taking 350 μL supernatant; and drying it with Nitrogen. Next, 120 μL 50% methanol was added to the dried samples, shaking at 1200 rpm for 20 min at 10 °C. Next, the samples were centrifuged at 18,000× *g* and 4 °C for 10 min, and 100 μL of supernatant was transferred to a new centrifuge tube for analysis.

#### 2.4.2. UPLC-MS/MS Analysis Parameters

Serum sweetener quantitation was performed using ultraperformance liquid chromatography coupled to a tandem mass spectrometry (UPLC-MS/MS) system (ACQUITY UPLC Xevo TQ-S, Waters Corp., Milford, MA, USA) according to previously published methods [20,21]. All chromatographic separations were performed with an UPLC HSS T3 (100 × 2.1 mm, 1.7 μm). The mobile phases consisted of 0.1% formic acid in water (A) and acetonitrile/methanol (7:3 + 0.1%FA, B) with a total flow rate of 0.4 mL/min. The gradient elution program was set as follows: 0–1 min (5% B), 1–1.8 min (5–20% B), 1.8–6 min (20–60% B), 6–7 min (60–100% B), 7–8 min (100% B), and 8–9 min (100–5% B). The cone and collision energy for each sweetener used the optimized settings.

For the mass spectrometer, the multiple reaction monitoring mode was used, with the electrospray ionization source in positive mode and negative mode. The capillary voltage was set at 2.0 kV in positive mode and 1.5 kV in negative mode. The desolvation temperature was set at 500 °C with a desolvation gas flow of 1000 L/Hr.

Quality control pools were constructed using equal volumes from all maternal samples and prepared for analysis as described above. QCs from each MS batch were first injected. The raw data files generated through UPLC-MS/MS were processed using the MassLynx software for peak integration, calibration, and quantification of each metabolite. A calibration curve was used to determine sweetener concentration. The testing service was provided by Metabo-Profile Inc., Shanghai, China.

### 2.5. Diagnosis and Definition

GDM was defined by the International Association of Diabetes and Pregnancy Study Groups criteria [22] as ≥1 of the following results: fasting ≥5.1 mmol/L; 1 h ≥ 10.0 mmol/L; and 2 h ≥ 8.5 mmol/L.

Abnormal glucose metabolism during pregnancy included elevated HbA1c, insulin resistance, and impaired insulin secretion. Elevated HbA1c was defined as ≥5.1% (the upper tertile of HbA1c). Insulin resistance was defined as ≤8.00 (the upper tertile of Matsuda ISI) or ≥2.4 (the lower tertile of HOMA-IR). Impaired insulin secretion was defined as ≤107.10 (the lower tertile of HOMA-β index).

Dyslipidemia during pregnancy included hypertriglyceridemia, hypercholesterolemia, hyper-LDL cholesterolemia, and hypo-HDL cholesterolemia. Few participants were diagnosed with dyslipidemia based on Williams Obstetrics 24th Edition. We applied the upper tertiles of lipids traits as cut-off criteria (the lower tertile for HDL-cholesterol). Hypertriglyceridemia was defined as a serum triglyceride level ≥1.9 mmol/L. Hypercholesterolemia was defined as a serum total cholesterol level ≥5.73 mmol/L. Hyper-LDL cholesterolemia was defined as a serum LDL- cholesterol levels ≥3.08 mmol/L. Hypo-HDL cholesterolemia was defined as a serum HDL- cholesterol ≤2.89 mmol/L.

### 2.6. Statistical Analysis

Continuous variables are shown as means ± SD or medians (interquartile range) for skewed variables. Categorized variables are presented as numbers (proportions). For a comparison of continuous variables, Student’s *t*-tests and Wilcoxon rank-sum tests were used. For a comparison of categorical data, chi-square tests or Fisher’s exact tests were used.

Spearman rank correlations were used to assess the relationships between serum aspartame and sucralose levels and maternal traits. Maternal traits included age at OGTT, gestational age at OGTT, pre-pregnancy BMI, MAP at OGTT, fasting, 1 h and 2 h plasma glucose, fasting, 1 h and 2 h insulin, HbA1c, Matsuda ISI, HOMA-IR, HOMA-β, triglycerides, total cholesterol, LDL cholesterol, and HDL cholesterol.

Multiple linear regression and robust linear regression models were used to evaluate the associations between serum aspartame and sucralose levels with glycemic and lipid traits during pregnancy. Robust linear regression is an alternative to the linear regression model in which divergent or influential values are weighted less heavily [23]. Adjusted cofactors included maternal age at OGTT, family history of diabetes (yes = 1, no = 0), MAP at OGTT, pre-pregnancy BMI, and parity (0, 1+).

Multiple logistic regression models were used to assess associations between higher serum aspartame and sucralose levels with abnormal glucose metabolism and dyslipidemia during pregnancy among all participants. Higher serum levels of aspartame and sucralose were defined as ≥0.06570 (nmol/L) and ≥0.4240 (nmol/L), respectively (the upper tertile of serum aspartame and sucralose levels). Adjusted cofactors included maternal age at OGTT, family history of diabetes (yes = 1, no = 0), MAP during OGTT, pre-pregnancy BMI, and parity (0, 1+). We reported odds ratios (ORs) and 95% CIs as measures of association. Results were considered significant associations if the 95% CIs did not contain 0.

Considering the metabolic differences between participants with and without GDM, logistic models were used to evaluate the interactions between higher aspartame/sucralose levels and GDM on abnormal glucose metabolism/dyslipidemia. Then, the associations between higher serum aspartame and sucralose levels with abnormal glucose metabolism/dyslipidemia were evaluated using multiple logistic regression models for participants with and without GDM.

A two-sided *p* value ≤ 0.05 was considered statistically significant. Comparisons of participant characteristics were carried out using SAS version 9.3 (SAS Institute, Cary, NC, USA).

## 3. Results

### 3.1. Distributions of Serum Aspartame and Sucralose Levels

We detected aspartame and sucralose levels in the blood of all participants at fasting state. The distribution of serum aspartame and sucralose levels was skewed. The median levels of serum aspartame and sucralose were 0.0563 (0.0459–0.0692) nmol/L and 0.3790 (0.3020–0.4740) nmol/L, respectively (Supplementary Figure S2).

### 3.2. Participants’ Characteristics

Demographic data are shown in Supplementary Table S1 for the 218 women (109 GDM and 109 non-GDM) who participated in the study and for whom serum aspartame and sucralose levels were available.

Serum aspartame and sucralose levels showed no significant differences in women with and without GDM (Supplementary Table S1 and Supplementary Figure S3). Women with GDM had higher levels of fasting, 1 h and 2 h plasma glucose, fasting and 2 h insulin, HbA1c, HOMA-IR, and triglycerides, and lower levels of Matsuda ISI (all *p* values < 0.05).

All participants were divided into higher and lower aspartame/sucralose groups according to the upper tertile of serum aspartame/sucralose levels. Table 1 shows the characteristics of participants with higher and lower aspartame/sucralose levels during pregnancy at OGTT. Maternal glycemic and lipid traits differed between the groups with higher and lower aspartame levels. Women with higher serum aspartame levels had higher levels of fasting, 1 h and 2 h insulin, HbA1c, HOMA-IR, total cholesterol, LDL cholesterol, and lower levels of Matsuda ISI (all *p* values < 0.05). Women with higher serum sucralose levels had lower HbA1c levels (*p* = 0.04).

### 3.3. Factors Associated with Serum Aspartame and Sucralose Levels

Although there were no differences between serum aspartame and sucralose levels in participants with and without GDM, we sought to identify the factors associated with serum aspartame and sucralose levels among all participants (ESM Table S2). Characteristics showing positive associations with serum aspartame levels included fasting, 1 h and 2 h insulin, HbA1c, HOMA-IR, total cholesterol, and LDL cholesterol. A negative association was found for Matsuda ISI. Only gestational age at OGTT showed a negative association with serum sucralose level.

### 3.4. Linear Associations between Serum Aspartame and Sucralose Levels and Glycemic and Lipid Traits

Multiple and robust linear regression models were used to determine the associations between serum aspartame and sucralose levels with maternal glycemic and lipid traits among all participants.

In the fully-adjusted linear regression models, serum aspartame levels were positively associated with total cholesterol and LDL cholesterol (coefficient ± SE: 0.2652 ± 0.1183, and 0.1250 ± 0.0582, and *p* = 0.026 and 0.033) and negatively associated with Matsuda ISI (coefficient ± SE: −0.3366 ± 0.1370, *p* = 0.015).

Similarly, the associations between serum aspartame levels and Matsuda ISI, total cholesterol, and LDL cholesterol persisted in the fully-adjusted robust linear regression models (coefficient ± SE: −0.3397 ± 0.1383, and 0.3052 ± 0.1415, and 0.1905 ± 0.0573, and *p* = 0.014, 0.031, and 0.0009, respectively). Emerging associations were for HOMA-IR and HOMA-β in the fully-adjusted robust linear regression models (coefficient ± SE: −0.3652 ± 0.1723 and 0.3482 ± 0.1590, and *p* = 0.034 and 0.029, respectively).

For serum sucralose levels, the only significant association was found for LDL cholesterol in the unadjusted linear regression model (coefficient ± SE: 0.1007 ± 0.0496, *p* = 0.044).

### 3.5. Categorized Serum Aspartame and Sucralose Levels Associated with Abnormal Glucose Metabolism and Dyslipidemia

**Figure 1:**
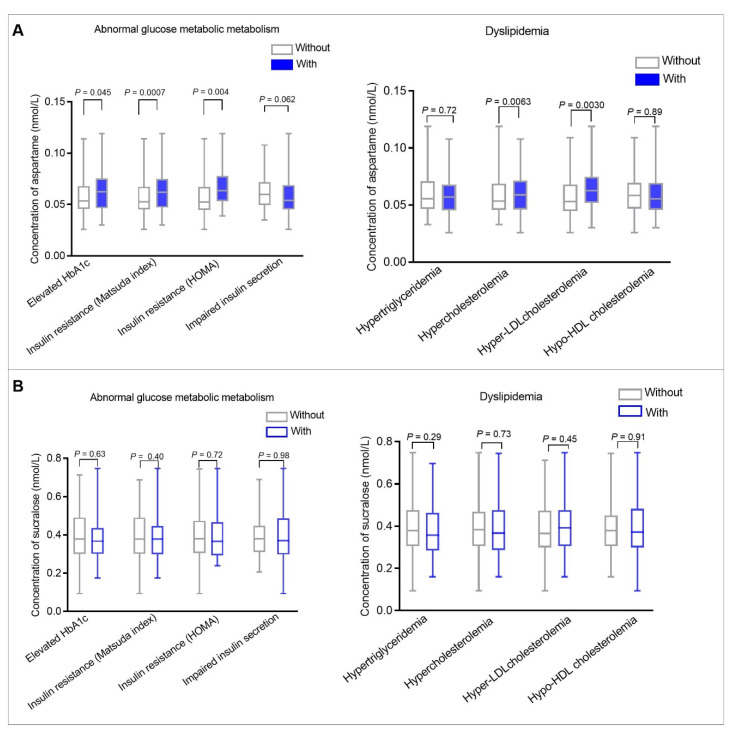
Box plot comparing serum aspartame (**A**) and sucralose (**B**) levels among all participants with and without abnormal glucose metabolism and dyslipidemia during pregnancy. The left panel (**A**) compares serum aspartame levels in women with and without abnormal glucose metabolism. The right panel (**A**) compares serum aspartame levels in women with and without dyslipidemia. The left panel (**B**) compares serum sucralose levels in women with and without abnormal glucose metabolism. The right panel (**B**) compares serum sucralose levels in women with and without dyslipidemia. Abnormal glucose metabolism during pregnancy included elevated HbA1c, insulin resistance, and impaired insulin secretion. Elevated HbA1c was defined as ≥5.1% (the upper tertile of HbA1c). Insulin resistance was defined as ≤8.00 (the upper tertile of Matsuda ISI) or ≥2.4 (the lower tertile of HOMA-IR). Impaired insulin secretion was defined as ≤107.10 (the lower tertile of HOMA-β index). Dyslipidemia during pregnancy included hypertriglyceridemia, hypercholesterolemia, hyper- LDL cholesterolemia, and hypo-HDL cholesterolemia. Hypertriglyceridemia was defined as ≥1.9 mmol/L (the upper tertile of triglycerides). Hypercholesterolemia was defined as ≥5.73 mmol/L (the upper tertile of total cholesterol). Hyper-LDL cholesterolemia was defined as ≥3.08 mmol/L (the upper tertile of LDL-cholesterol). Hypo-HDL cholesterolemia was defined as ≤2.89 mmol/L (the lower tertile of HDL-cholesterol). Wilcoxon rank-sum tests were used to compare serum aspartame and sucralose levels among all participants with and without abnormal glucose metabolism and dyslipidemia. *p* values less than 0.05 were considered statistically significant.

In the linear regression models, glycemic and lipid traits showed significant associations with serum aspartame levels. Subsequent analyses compared serum aspartame and sucralose levels in participants with and without abnormal glucose metabolism/dyslipidemia (Figure 1, Supplementary Figure S4, ESM Tables S3 and S4). Figure 1 shows that among all participants, women with elevated HbA1c, insulin resistance (defined according to Matsuda ISI and HOMA-IR), hypercholesterolemia, and hyper-LDL cholesterolemia had higher serum aspartame levels compared to those without. No differences were found for impaired insulin secretion, hypertriglyceridemia, or hypo-HDL cholesterolemia. Serum sucralose levels showed no differences between women with abnormal glucose metabolism/dyslipidemia and those without.

**Figure 2:**
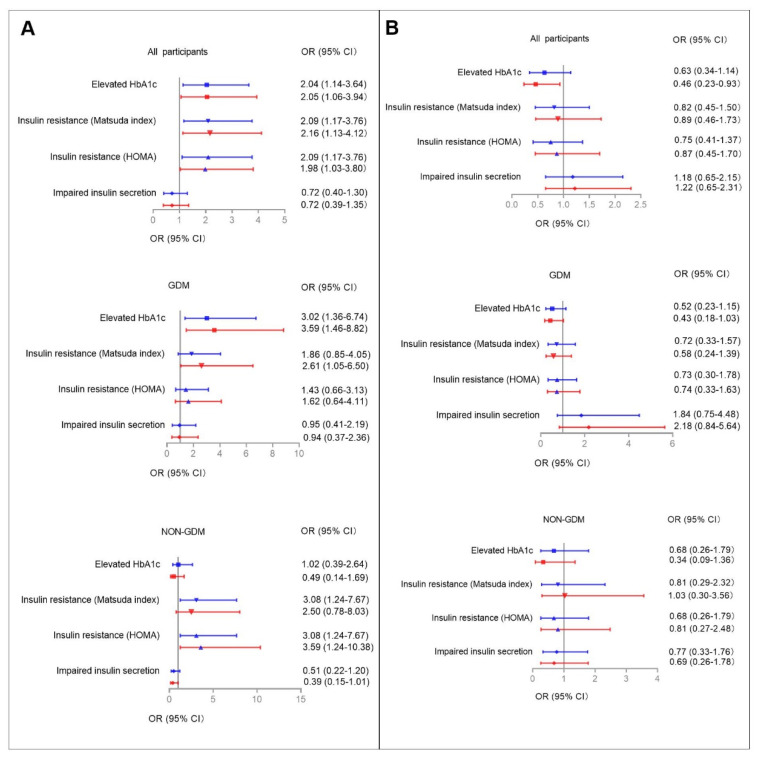
The associations between higher serum aspartame (**A**) and sucralose (**B**) levels with abnormal glucose metabolism during pregnancy. Higher serum aspartame and sucralose levels were defined as ≥0.0657 (nmol/L) and ≥0.4240 (nmol/L), respectively. Multiple logistic regression models were used to determine the associations between higher serum aspartame and sucralose levels with abnormal glucose metabolism during pregnancy among all participants, and among those with and without GDM, respectively. Adjusted cofactors included maternal age, family history of diabetes, maternal MAP at OGTT, pre-pregnancy BMI, and parity (0, 1+). Blue and red lines indicate ORs (95% CIs) before and after full adjustments, respectively. Reference: Women lower aspartame/sucralose levels among all participants, and those with and without GDM, respectively. Results were considered significant associations if the 95% CIs did not contain 0.

**Figure 3:**
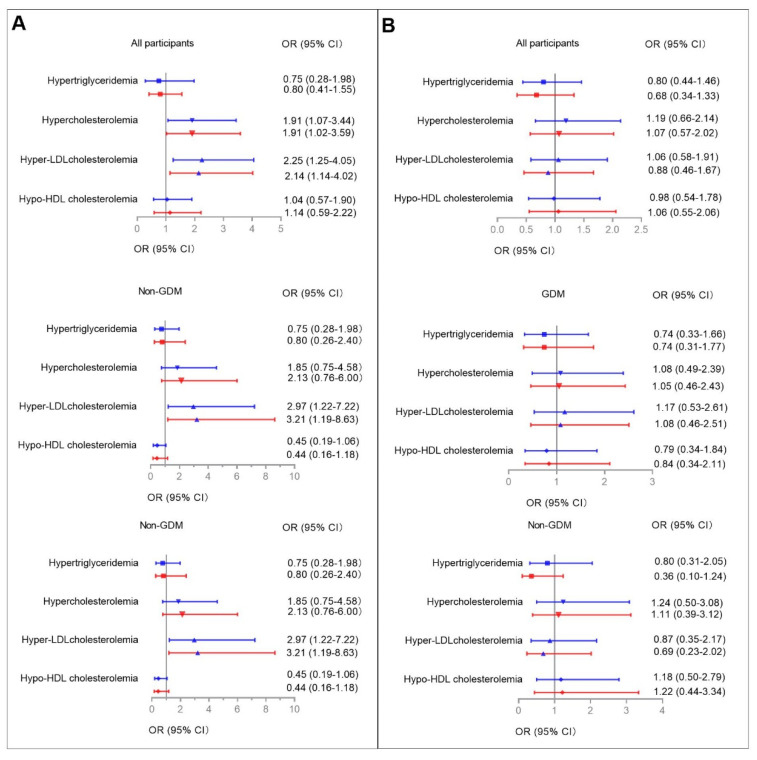
Associations between higher serum aspartame (**A**) and sucralose (**B**) levels with dyslipidemia during pregnancy. Multiple logistic regression models were used to determine the associations between higher serum aspartame and sucralose levels with dyslipidemia among all participants and among those with and without GDM, respectively. Covariates for fully adjusted models are as stated in the legend for Figure 2. Blue and red lines indicate ORs (95% CIs) before and after full adjustments, respectively. Reference: Women without lower serum aspartame/sucralose levels among all participants, and among those with and without GDM, respectively. Results were considered significant associations if the 95% CIs did not contain 0.

Multiple logistic regression models were used to determine the associations between higher serum aspartame and sucralose levels with abnormal glucose metabolism/dyslipidemia during pregnancy among all participants (Figure 2 and Figure 3, Supplementary Tables S5 and S6). Higher serum aspartame levels were significantly associated with abnormal glucose metabolism/dyslipidemia. The ORs in the unadjusted logistic models were 2.04 (95% CI: 1.14–3.64) for elevated HbA1c, 2.09 (95% CI: 1.17–3.76) for insulin resistance according to both Matsuda ISI and HOMA-IR, 1.91 (95% CI: 1.07–3.44) for hypercholesterolemia, and 2.25 (95% CI: 1.25–4.05) for hyper-LDL cholesterolemia. The ORs in the fully-adjusted logistic models were 2.05 (95% CI: 1.06–3.94) for elevated HbA1c, 2.16 (95% CI: 1.13–4.12) and 1.98 (95% CI: 1.03–3.80) for insulin resistance defined by Matsuda ISI and HOMA-IR, 1.91 (95% CI: 1.02–3.59) for hypercholesterolemia, and 2.14 (95% CI: 1.14–4.02) for hyper-LDL cholesterolemia.

Serum sucralose levels were not associated with abnormal glucose metabolism or dyslipidemia, except for elevated HbA1c. In the unadjusted model, the OR was 0.63 (95% CI: 0.34–1.14) for elevated HbA1c, whereas full adjustment made the associations significant (OR: 0.46, 95% CI: 0.23–0.93).

### 3.6. Categorized Serum Aspartame and Sucralose Levels Associated with Abnormal Glucose Metabolism/Dyslipidemia in GDM and Non-GDM Groups

Given the metabolic differences between participants with and without GDM, we then used logistic models to examine the interactions between categorized serum aspartame and sucralose levels and GDM on abnormal glucose metabolism/dyslipidemia (Figure 2 and Figure 3, Supplementary Figure S4, ESM Tables S3 and S4). The relevant interactions were found between categorized serum aspartame levels and abnormal glucose metabolism/dyslipidemia.

In the GDM group, higher serum aspartame levels were found in those with elevated HbA1c, insulin resistance defined according to Matsuda ISI, and hypercholesterolemia compared to those with lower serum asparatame levels. In the fully-adjusted logistic regression models, the significant associations were for elevated HbA1c (OR: 3.59, 95% CI: 1.46–8.82), insulin resistance defined according to Matsuda ISI (OR: 2.61, 95% CI: 1.05–6.50), and hypo-HDL cholesterolemia (OR: 3.24, 95% CI: 1.15–9.16), respectively.

In the non-GDM group, higher serum levels of aspartame were found in those with insulin resistance defined according to Matsuda ISI and hyper-LDL cholesterolemia. In the fully-adjusted logistic regression models, the significant associations were for insulin resistance according to HOMA-IR criteria and hyper-LDL cholesterolemia with ORs of 3.59 (95% CI: 1.24–10.38) and 3.21 (95% CI: 1.19–8.63).

No associations were found between serum levels of sucralose and either glucose metabolism or lipid profiles in the GDM and non-GDM groups, respectively.

## 4. Discussion

Our results reported the quantitative detection of serum aspartame and sucralose in pregnant women. Serum aspartame levels were positively associated with maternal insulin resistance index, total cholesterol, and LDL cholesterol during pregnancy. These associations were independent of possible confounders, including age, pre-pregnancy BMI, family history of diabetes, parity, and MAP at OGTT. In the GDM group, the significant associations between higher serum aspartame levels and elevated HbA1c, insulin resistance, and hypo-HDL cholesterolemia persisted, while positive associations were found between higher serum aspartame levels and insulin resistance and hyper-LDL cholesterolemia in the non-GDM group. Moreover, serum sucralose levels were not associated with glucose and lipids profiles except for HbA1c.

NNS cannot be synthesized in the body. Upon ingestion, aspartame is metabolized quickly in the intestine, including aspartic acid, phenylalanine, and methanol, while sucralose is absorbed and then mostly excreted in urine and feces [24]. Therefore, NNS were previously thought to be undetectable in circulation. With the development of novel testing techniques, some NNS were recently detected in vivo, including sucralose, acesulfame, saccharin, and cyclamate [9,10]. Our findings may be the first report of circulating aspartame and sucralose levels in pregnant women. The relatively low concentration observed in this study was consistent with the metabolism of the two NNS in vivo. Despite the metabolism of NNS in the intestine, another possible pathway into the human body for NNS is absorption through the mouth. The threshold of substance absorbed by the oral mucosa is 800 Da, which is much greater than the molecular weight of aspartame and sucralose (294.3 and 397.634 Da). However, this possibility requires further investigation.

Even at comparatively low concentrations, serum aspartame levels showed significant associations with maternal glycemic traits in several different statistical models in the present study. Aspartame and its degradation products were reported to cause insulin resistance and abnormal glucose metabolism in non-gravid animals [3,5]. Several mechanisms could be involved in this phenomenon, primarily including energy imbalance, alteration of the gut microbiota, disruption of neuroendocrine balance, and oxidant/antioxidant activities [5,24]. More specifically, the decrease in energy expenditure after feeding mice with aspartame (0.5 mg/g) may be due to the arcuate nucleus of the hypothalamus [25]. As aspartame is a chemical stressor of the brain HPA, aspartame supplementation in rodent models can elevate corticosterone levels [26], increase muscarinic receptor density [27], and inhibit serotonin, noradrenaline, and dopamine in the brain [28], consequently resulting in abnormal glucose metabolism. Additionally, aspartame ingestion (240 mg/kg body weight/day for 2 months) resulted in liver injury via antioxidant status [4]. Notably, human-based evidence, from available randomized controlled trials (RCTs) and observational studies, remains scarce and inconclusive [24]. A RCT study reported that lean humans consuming aspartame for 12 weeks did not show glucose fluctuations or weight gain [29]. Conversely, L Kuk, et.al., found in the NHANES III survey that the consumption of aspartame was associated with greater obesity-related impairments in glucose tolerance [30]. Consistent with this result, we found that circulating aspartame concentrations had a positive association with maternal insulin resistance in women with GDM during pregnancy.

Few studies have examined the effects of aspartame supplementation on maternal glucose and insulin levels during pregnancy. Additionally, previous work primarily focused on offspring outcomes, but not on maternal metabolism. In this study, we reported that maternal serum aspartame levels were positively associated with HbA1c, fasting insulin, 1 h and 2 h insulin during pregnancy at OGTT. The observational findings remained consistent with previous results from animal and human studies. Rats receiving aspartame increased their food intake by 1.1-fold during pregnancy [31]. In a population-based study, Meghan B. Aza et al., found that mothers reporting daily NNS beverage consumption had higher BMIs during pregnancy [1]. The underlying mechanisms for the associations between aspartame and disorders of glucose during pregnancy, however, remain unclear. Whether these mechanisms are comparable to those with non-gravid status requires further study.

Serum aspartame levels were significantly associated with maternal lipid metabolism in our study. Whether aspartame consumption could induce lipid imbalance, however, remains unknown. Early-years studies from diverse ancestries reported that a single-dose or short-period consumption of aspartame did not alter lipids profiles in patients with diabetes [32,33]. Recently, more research on rodent models raised opposite conclusions, indicating the emergence of lipid profile disorders [4,34] after aspartame intake. The underlying mechanisms likely include oxidant/antioxidant imbalance, systematic inflammation, and the cortisol pathway [35].

Serum sucralose levels were negatively associated with HbA1c in our study. Likewise, some previous studies on the impacts of sucralose intake on human glucose metabolism reported beneficial effects [6], whereas others found harmful [36] or minimal effects [37,38,39]. One possible interpretation of these contradictions could be the dose-dependent effects of sucralose exposure on glucose metabolism. Our previous research in HFD rats found that 0.78 mM sucralose consumption for a month improved glucose tolerance at all the time points of OGTT compared to the control. However, a 0.54 mM-dose decreased blood glucose levels at only 120 min [34]. This result suggests that an optimal dose of sucralose could be applied for better glucose control. Additionally, sucralose was demonstrated to affect glucose metabolism, in part, through the human genetic polymorphism of type 1 taste receptor 3 (T1R3) and its downstream pathways [40]. Genetic studies have revealed that T1R3 contributes to individual differences in sweet sensitivities [41].

There are several limitations of this study. Firstly, the cross-sectional design of this study failed to establish causality and therefore the associations observed in the study may be due to reverse causation. Secondly, the small sample size might limit statistical power. Thirdly, dietary intake information on aspartame and sucralose was not collected. Therefore, there is a high likelihood that those with metabolic abnormalities, including obesity and overweight may be more inclined to consume NNS as added sugar replacers. Nevertheless, diet may not be the only source of NNS in the human body as mentioned previously. Compared to the NNS intake questionnaire, the measurement of circulating NNS could be an alternative way to quantify NNS exposure from different sources in life (i.e., industrial food and drinking water). Fourthly, the current study captures only a portion of the possible factors of perpetuated glucose and lipid metabolism. Therefore, additional studies would be needed to explore the potential contributions of additional factors, such as dietary habits, regular leisure-time physical exercises, and socioeconomic status. Lastly, the study was nested in a cohort study in Shanghai, China. Considering the diverse dietary habit, these associations should be confirmed in those from other ancestries.

## 5. Conclusions

To conclude, maternal serum aspartame levels were significantly associated with impaired glucose and lipids metabolism during pregnancy at OGTT, while serum sucralose levels were negatively associated with HbA1c. More robust prospective studies are warranted to confirm the cross-sectional associations observed in this study.

## Figures and Tables

**Table 1:** Characteristics of participants with higher and lower serum levels of aspartame/sucralose.

Trait (Units)	Serum Aspartame Levels			Serum Sucralose Levels		
	Lower (*n* = 144)	Higher (*n* = 74)	*p*	Lower (*n* = 143)	Higher (*n* = 75)	*p*
Aspartame/sucralose levels (nmol/L)	0.0495 (0.0432–0.0560)	0.0765 (0.0692–0.0841)	-	0.32 (0.28–0.37)	0.51 (0.46–0.57)	-
Age at OGTT (years)	30.1 ± 4.3	29.9 ± 4.4	0.70	29.9 ± 4.3	30.4 ± 4.3	0.44
Gestational age at OGTT (weeks)	25.0 (25.0–26.0)	25.0 (25.0–26.0)	0.70	25.0 (25.0–26.0)	25.0 (24.0–26.0)	0.13
Pre-pregnancy BMI (kg/m^2^)	22.38 ± 3.34	22.88 ± 3.42	0.30	22.62 ± 3.43	22.52 ± 3.46	0.75
BMI (kg/m^2^)	25.07 ± 3.66	25.51 ± 3.46	0.39	25.31 ± 3.54	25.04 ± 3.70	0.61
MAP (mmHg)	87.1 ± 8.4	89.4 ± 9.3	0.073	88.2 ± 9.0	87.4 ± 8.3	0.56
FPG (mmol/L)	4.57 ± 0.48	4.67 ± 0.49	0.13	4.64 ± 0.48	4.53 ± 0.49	0.12
1 h plasma glucose levels (mmol/L)	8.39 ± 1.87	8.72 ± 1.77	0.20	8.57 ± 1.83	8.36 ± 1.87	0.42
2 h plasma glucose levels (mmol/L)	7.44 ± 1.62	7.71 ± 1.68	0.26	7.56 ± 1.61	7.49 ± 1.71	0.79
Fasting insulin levels (mmol/L)	41.54 (27.29–61.55)	56.63 (34.57–77.22)	0.0087	50.87 (29.62–70.76)	42.20 (29.36–67.76)	0.34
1 h insulin levels (mmol/L)	334.15 (208.45–538.55)	402.00 (319.40–534.50)	0.026	368.50 (251.70–540.70)	345.00 (229.50–533.60)	0.58
2 h insulin levels (mmol/L)	314.85 (235.15–473.95)	449.65 (293.20–582.00)	0.0092	366.60 (240.70–522.10)	346.00 (235.80–519.80)	0.95
HbA1c (%)	4.89 ± 0.37	5.03 ± 0.37	0.014	4.98 ± 0.36	4.87 ± 0.40	0.04
HbA1c (mmol/mol)	29.99 ± 4.06	31.43 ± 4.05	0.014	30.90 ± 3.93	29.68 ± 4.34	0.04
Matsuda ISI	5.99 (4.00–8.98)	4.44 (3.56–6.93)	0.004	5.44 (3.75–7.49)	5.61 (3.95–9.24)	0.46
HOMA-IR	1.40 (0.86–2.21)	1.96 (1.10–2.73)	0.0095	1.70 (0.96–2.53)	1.44 (0.94–2.29)	0.30
HOMA-β	144.64 (103.52–195.09)	155.01 (114.68–253.42)	0.14	140.24 (105.41–216.83)	152.07 (113.43–195.77)	0.56
Triglycerides (mmol/L)	1.58 (1.27–2.07)	1.74 (1.37–2.19)	0.21	1.65 (1.31–2.09)	1.60 (1.30–2.10)	0.93
Total cholesterol (mmol/L)	5.25 ± 0.84	5.55 ± 0.80	0.014	5.33 ± 0.82	5.40 ± 0.88	0.56
LDL cholesterol (mmol/L)	2.70 (2.35–3.13)	3.01 (2.64–3.48)	0.0035	2.78 (2.36–3.24)	2.93 (2.46–3.41)	0.27
HDL cholesterol (mmol/L)	2.65 ± 0.56	2.67 ± 0.51	0.86	2.66 ± 0.54	2.65 ± 0.55	0.92
Family history of diabetes, No. (%)	9 (6.3)	7 (9.5)	0.39	8 (5.6)	8 (10.7)	0.17
GDM, No. (%)	76 (46.5)	42 (56.8)	0.15	69 (48.3)	40 (53.3)	0.48

Data are given as the median (interquartile range) for skewed variables, or as the number (proportion) for categorical variables. For comparisons of continuous variables, Student’s *t*-tests and Wilcoxon rank-sum tests were used. For comparisons of categorical variables, the chi-square tests or Fisher’s exact tests were used. *p* values less than 0.05 were considered statistically significant. Higher serum aspartame and sucralose levels were defined as ≥0.0657 and ≥0.4240 nmol/L, respectively (the upper tertile of serum aspartame and sucralose levels). Abbreviations: OGTT: oral glucose tolerance test, MAP: mean arterial pressure, FPG: fasting plasma glucose, ISI: insulin sensitivity index, HOMA-IR, homeostasis model assessment for insulin resistance, GDM: gestational diabetes mellitus, LDL: low-density lipoprotein, HDL: high-density lipoprotein.

## Data Availability

Data and codes used for analyses will be made available by the authors upon request.

## Supplementary Materials

The following supporting information can be downloaded at: https://www.mdpi.com/article/10.3390/nu14235001/s1. Figure S1. Flow diagram for the enrollment of the study. Figure S2. Violin plots presenting the distributions of serum aspartame (A) and sucralose (B) levels among all participants during pregnancy. Figure S3. Box plot comparing serum aspartame (A) and sucralose (B) levels among participants with and without GDM during pregnancy. Figure S4. Box plot comparing serum aspartame (A) and sucralose (B) levels among participants with and without abnormal glucose metabolism/dyslipidemia in the GDM and non-GDM groups. Table S1. Participants’ characteristics in the GDM and non-GDM groups. Table S2. Spearman correlation analysis between serum aspartame and sucralose levels and maternal characteristics. Table S3. Linear regression and robust regression models to identify the associations between maternal serum aspartame levels with glycemic and lipids traits. Table S4. Linear regression and robust regression models to identify the associations between maternal serum sucralose levels with glycemic and lipids traits. Table S5. Logistic regression models to identify the associations between higher se rum aspartame levels with abnormal glucose metabolism and dyslipidemia. Table S6. Logistic regression models to identify the associations between higher serum sucralose levels with abnormal glucose metabolism and dyslipidemia.
