# Supplementary material for: The Associations between Maternal Serum Aspartame and Sucralose and Metabolic Health during Pregnancy
Source: Nutrients. 2022 Nov 24;14(23):5001. doi: 10.3390/nu14235001 (PMC9740469; doi:10.3390/nu14235001)
Supplement: Supplementary file 1 [file nutrients-14-05001-s001.zip › ESM FIG_version_9.pdf]

Supplementary Figure S1.

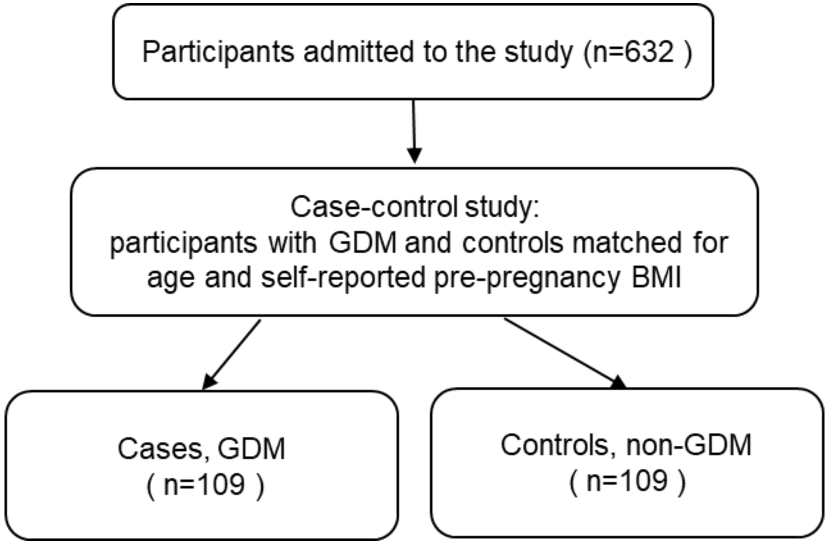

Supplementary Figure S1. Flow diagram for the enrollment of the study. All participants with GDM were selected as cases from 632 pregnant women, except for 4 women missing blood samples. The final analysis included 109 cases and 109 controls matched for age and self-reported pre-pregnancy BMI. Abbreviation: GDM, Gestational diabetes mellitus.

Supplementary Figure S2.

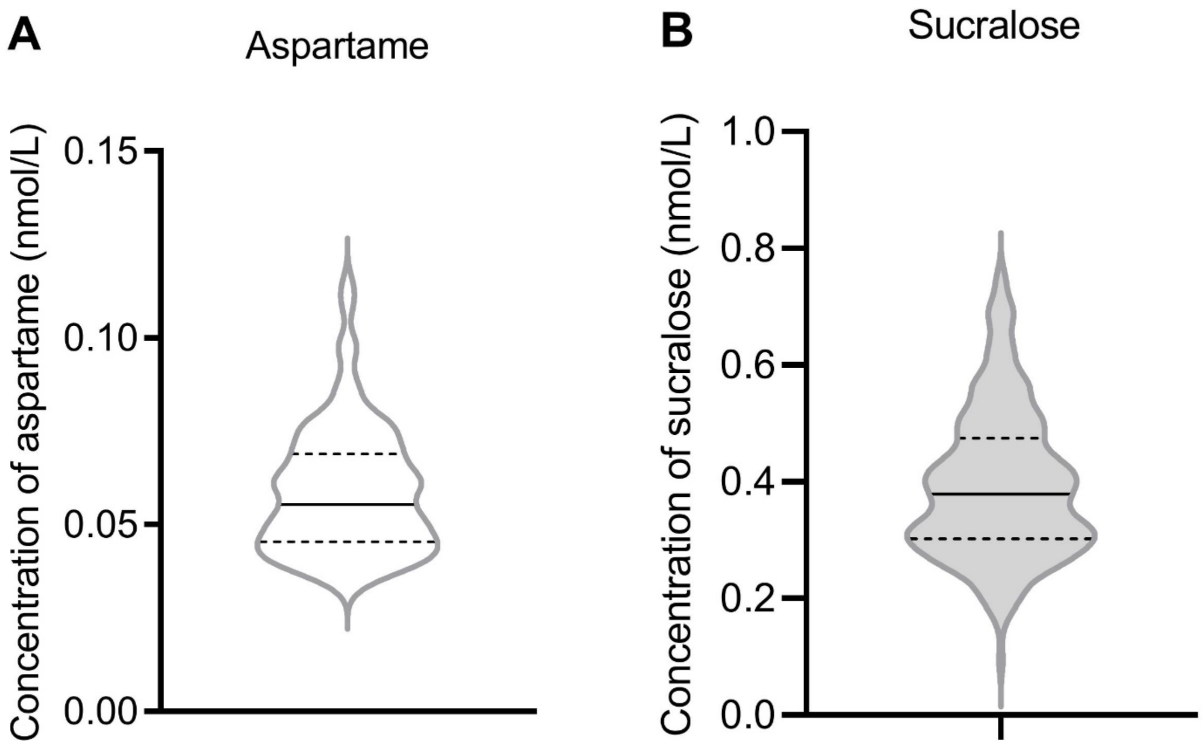

Supplementary Figure S2. Violin plots presenting the distributions of serum aspartame (A) and sucralose (B) levels among all participants during pregnancy. The median levels of serum aspartame and sucralose were 0.0563 (0.0459-0.0692) nmol/L and 0.3790 (0.3020-0.4740) nmol/L, respectively.

Supplementary Figure S3.

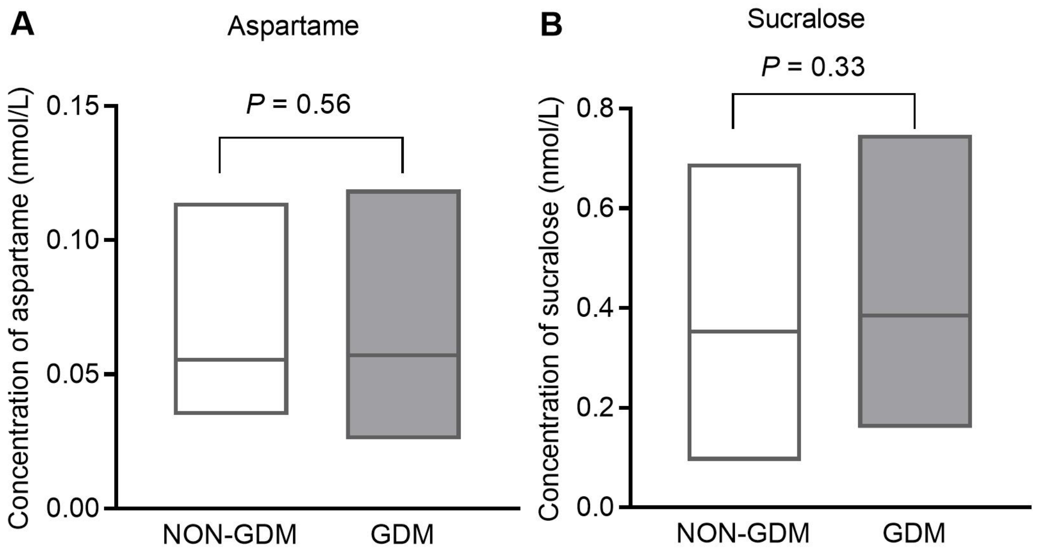

Supplementary Figure S3. Box plot comparing serum aspartame (A) and sucralose (B) levels among participants with and without GDM during pregnancy. Wilcoxon rank-sum tests were used to compare serum aspartame and sucralose levels among participants with and without GDM. *P* values less than 0.05 were considered statistically significant.

Supplementary Figure S4.

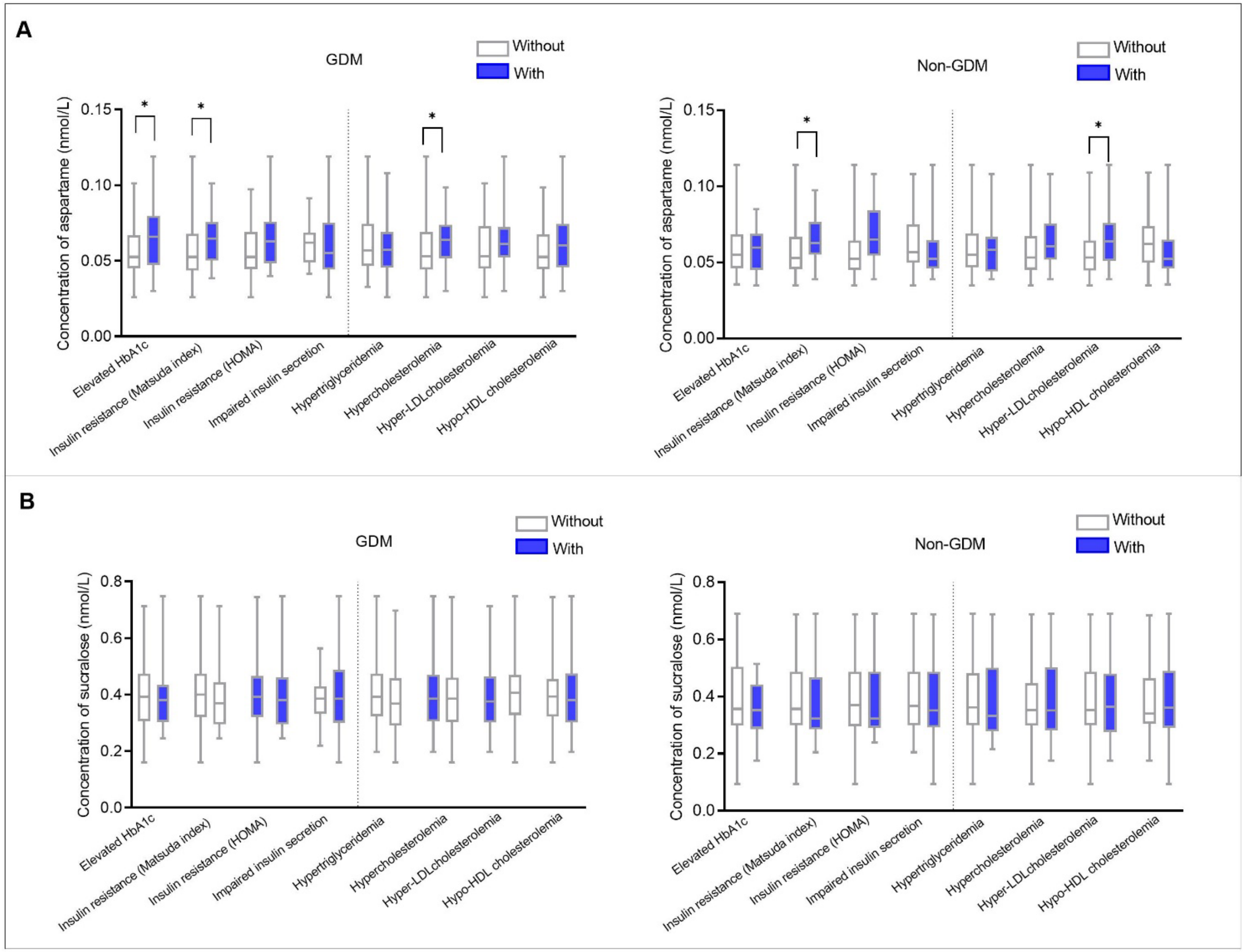

Supplementary Figure S4. Box plot comparing serum aspartame (A) and sucralose (B) levels among participants with and without abnormal glucose metabolism/dyslipidemia in the GDM and non-GDM groups. The upper panel (A) compares serum aspartame levels among participants with and without abnormal glucose and dyslipidemia in the GDM and non-GDM groups. The lower panel (B) is for comparing serum sucralose levels among participants with and without abnormal glucose and dyslipidemia in the GDM and non-GDM groups.

Abnormal glucose metabolism during pregnancy included elevated HbA1c, insulin resistance, and impaired insulin secretion. Elevated HbA1c was defined as ≥5.1% (the upper tertile of HbA1c). Insulin resistance was defined as ≤8.00 (the upper tertile of Matsuda insulin sensitivity index derived from OGTT during pregnancy) or ≥2.4 (the lower tertile of HOMA-IR). Impaired insulin secretion was defined as ≤107.10 (the lower tertile of HOMA-β index).

Dyslipidemia during pregnancy included hypertri-glyceridemia, hypercholesterolemia, hyper- LDL cholesterolemia, and hypo-HDL cholesterolemia. Hypertriglyceridemia was defined as ≥1.9 mmol/L (the upper tertile of triglycerides). Hypercho-lesterolemia was defined as ≥5.73 mmol/L (the upper tertile of total cholesterol). Hyper-LDL cholesterolemia was defined as ≥3.08 mmol/L (the upper tertile of LDL-cholesterol). Hypo-HDL cholesterolemia was defined as ≤2.89 mmol/L (the lower tertile of HDL-cholesterol).

Wilcoxon rank-sum tests were used to compare serum aspartame and sucralose levels with and without abnormal glucose and dyslipidemia among all participants and those with and without GDM, respectively. *P* values less than 0.05 (asterisk superscript) were considered statistically significant.
